# Perfectionism and college students’ psychological adaptation: the mediating and moderating role of physical education satisfaction

**DOI:** 10.3389/fpsyg.2026.1836501

**Published:** 2026-07-08

**Authors:** XuPeng Zhang, ChangNan Xu, ZhenXiang Guo, MengBiao Cai, Chen Yang

**Affiliations:** 1 Department of Physical Education, Nanjing University of Aeronautics and Astronautics, Nanjing, China; 2 Sports Coaching College, Beijing Sport University, Beijing, China; 3 Department of Physical Education, Nanjing University, Nanjing, China

**Keywords:** college students, expressive suppression, gender differences, perfectionism, physical education satisfaction, psychological adaptation

## Abstract

**Purpose:**

This study examined how perfectionism (personal standards, concern over mistakes) influences college students’ psychological adaptation through physical education (PE) satisfaction, and the moderating roles of expressive suppression and gender.

**Methods:**

Chinese college students (*N* = 1,652; 925 males; aged 17–24) completed measures of perfectionism, emotion regulation, PE satisfaction, subjective vitality, subjective well-being, and academic burnout. Moderated mediation models were tested.

**Results:**

PE satisfaction significantly mediated effects of personal standards (vitality: beta = 0.375; well-being: beta = 0.362; both *p* < 0.001) and concern over mistakes (vitality: beta = 0.215; well-being: beta = 0.203; both *p* < 0.001) on positive adaptation, but negligibly mediated the concern over mistakes-burnout link (beta = 0.077). Expressive suppression produced differential moderating patterns: a dual weakening effect on the personal standards pathway, simultaneously attenuating the perfectionism-to-satisfaction stage (beta = −0.103, *p* < 0.001) and the satisfaction-to-well-being stage (beta = −0.078, *p* < 0.001), reducing indirect effects by 55.5%; and an emotional blunting effect on the concern over mistakes pathway, selectively weakening only the satisfaction-to-well-being link (beta = −0.081, *p* < 0.001) while sparing the initial perfectionism-to-satisfaction stage. Gender moderated only the concern over mistakes pathway (beta = 0.110, *p* = 0.044), with significant negative indirect effects exclusive to males.

**Conclusion:**

PE satisfaction serves as a gain system that amplifies adaptive traits yet cannot buffer inherent burnout tendencies. Expressive suppression effects vary across personality dimensions, challenging the dichotomous view of emotion regulation strategies. Gender differences reflect social role expectations rather than essential psychological disparities.

## Introduction

College student mental health has become a pivotal issue in global public health research. In recent years, the rising incidence of anxiety (Hang et al., 2026), academic burnout (Li et al., 2026), and declining subjective well-being (Liu et al., 2024) within higher education settings has spurred extensive investigation into both risk and protective factors. Among the numerous personality traits influencing psychological adaptation, perfectionism has garnered sustained attention due to its unique dual nature. Since Frost et al. conceptualized multidimensional perfectionism (Frost et al., 1990), researchers have generally distinguished two core dimensions: personal standards and concern over mistakes (Berna et al., 2002; Hill et al., 2020; Lavrijsen et al., 2021). Personal standards reflect an individual’s tendency to set high goals and strive for excellence, often viewed as a relatively positive aspect of perfectionism (Stoeber and Otto, 2006). Conversely, concern over mistakes reflects a fear of failure and excessive worry about others’ evaluations, considered the core maladaptive component (Mofield et al., 2016; Stoeber, 2018b). Meta-analytic evidence indicates that concern over mistakes is consistently associated with psychological distress indicators such as anxiety, depression, and academic burnout, whereas the role of personal standards is more complex, sometimes predicting psychological resilience and other times linking to vulnerability depending on contextual factors (Hill and Curran, 2016). These inconsistent findings suggest that perfectionism’s impact on psychological adaptation may not be a direct linear relationship but rather mediated through specific mechanisms. For instance, recent evidence from nursing education shows that maladaptive perfectionism influences interpersonal sensitivity through the sequential mediators of coping style and subjective well-being (Lu et al., 2024), underscoring the need to identify domain-specific pathways. In other words, perfectionism can represent a “crisis” impairing mental health or, under suitable conditions, transform into an “opportunity” fostering growth.

The importance of situational factors in perfectionism research is increasingly recognized (Tengti, 2025; Goodwin et al., 2025; Hill, 2026). One noteworthy context is physical education (PE) class—an institutionalized form of physical activity within higher education. The beneficial effects of physical exercise on mental health are well-established, with regular physical activity effectively alleviating anxiety (Munro et al., 2026) and depressive symptoms, and enhancing individuals’ subjective vitality and overall well-being (Munro et al., 2026; Teychenne et al., 2026). However, while existing research confirms the positive effects of PE participation on mental health, most studies focus on the main effect of participation versus non-participation (Ma and Mumtaz, 2025; Ooms et al., 2025). There is relatively limited mechanistic exploration of how individual differences shape the PE experience and how this experience translates into psychological adaptation outcomes (Battaglia et al., 2025; Yıldız and Çalı, 2026; Zhang and Han, 2026). Unlike academic courses, which are typically private or text-based, PE classes involve embodied public performance, immediate physical feedback, and direct social comparison (Liu, 2025; Margas et al., 2006). This unique context may amplify the psychological effects of the two perfectionism dimensions: For students high in concern over mistakes, the discrepancy between ideal and actual physical performance is readily apparent, potentially triggering their fear of evaluation (Ruiz et al., 2023; Short and Mazmanian, 2013; Triguero Martín et al., 2024). For students with high personal standards, achieving goals in PE may yield a stronger sense of competence (Pineda-Espejel, 2025; Yakushina et al., 2023). Embodied cognition theory supports this perspective, emphasizing that bodily experiences fundamentally shape psychological states (Mendes, 2026). Therefore, students’ subjective evaluation of PE—namely, PE satisfaction—may be a key mediator linking the two perfectionism dimensions to psychological adaptation. The embodied and publicly evaluative nature of PE classes may activate perfectionistic concerns in ways that private academic settings do not. Beyond PE, similar mechanisms have been observed in other high-stakes educational contexts: perfectionism predicts relative deprivation via interpersonal sensitivity, with resilience moderating both the initial and direct pathways (Ding et al., 2024a). This pattern reinforces the plausibility of a moderated mediation model in PE settings.

Self-determination theory (SDT) provides a systematic framework for understanding this mediating mechanism. SDT posits that the satisfaction of basic psychological needs-competence, autonomy, and relatedness-within specific domains forms the foundation for overall well-being (Ryan and Deci, 2000). In the PE context, course satisfaction subjectively reflects the degree to which these needs are met during physical activity (Langøy et al., 2024; Xu et al., 2025). The theoretical rationale for PE satisfaction as a mediator is threefold. First, PE class constitutes a unique embodied setting where competence is immediately and publicly validated-bodily performance is directly observable by peers, making the satisfaction of competence needs more salient than in traditional academic settings (Pineda-Espejel et al., 2023). Second, the autonomy-supportive structure of PE courses, wherein students exercise choice over movement strategies and effort levels, can differentially meet the autonomy needs of students with varying perfectionism profiles (Langøy et al., 2024). Third, the inherently social and collaborative nature of PE activities makes relatedness need satisfaction a critical component of course satisfaction (Vazou et al., 2019). When these three basic needs are satisfied in PE, domain-specific satisfaction can generate spillover effects, influencing overall psychological functioning (Ilies et al., 2009). These spillover effects can be examined through different facets of psychological adaptation. On one hand, when students experience competence satisfaction in PE, this positive experience may translate into daily feelings of vitality and well-being-the former reflecting perceived energy and aliveness (Pineda-Espejel et al., 2023; Ryan and Frederick, 1997), and the latter representing individuals’ global cognitive evaluation of their life quality (Lyubomirsky and Lepper, 1999; Miklitz et al., 2024). On the other hand, if the PE experience fails to satisfy basic psychological needs, particularly for students high in concern over mistakes, this frustration may exacerbate exhaustion and disengagement in academic contexts, i.e., academic burnout (Bochniarz et al., 2025; González-Cutre et al., 2025; Schaufeli et al., 2002). Academic burnout, as a maladaptive outcome, has been linked to perfectionism through similar need-frustration processes. In undergraduate nursing students, for example, self-efficacy mediates the perfectionism-academic procrastination relationship, with resilience serving as a buffering moderator (Huang et al., 2023); moreover, resilience and positive coping styles significantly moderate the link between maladaptive perfectionism and procrastination (Huang et al., 2022a). These findings suggest that domain-specific satisfaction (or its absence) may be a key transmission mechanism—a hypothesis we extend to the PE context. Thus, simultaneously examining subjective vitality, subjective well-being, and academic burnout helps comprehensively reveal the transmission role of PE satisfaction between perfectionism and psychological adaptation, covering both positive and negative aspects.

Furthermore, the strength of the pathways through which the two perfectionism dimensions influence psychological adaptation via PE satisfaction may vary depending on individuals’ emotion regulation strategies (Cao and Bao, 2026; Pineda-Espejel, 2025). Emotion regulation theory distinguishes between cognitive reappraisal and expressive suppression. Expressive suppression, as a response-focused strategy involving the inhibition of ongoing emotional expression, can isolate individuals from positive feedback experiences and consume cognitive resources (Cutuli, 2014; Gross, 1998). The PE context is uniquely relevant to expressive suppression for several reasons. Physical activities inherently generate immediate emotional responses-triumph after a successful performance, frustration after a mistake, joy from team collaboration-and these emotions are typically expected to be expressed through facial expressions, body language, and vocalizations (Fernandes and Tone, 2021). When students habitually suppress these natural emotional expressions during PE, two detrimental processes may unfold: First, the suppression of positive emotions (e.g., pride after skill mastery) may prevent the full internalization of competence experiences, reducing the satisfaction derived from PE participation (Aldao et al., 2010). Second, the cognitive resources consumed by chronic suppression may deplete the attentional capacity needed to benefit from the autonomy-supportive features of PE, thereby interfering with need satisfaction (Fernandes and Tone, 2021). Perfectionists often suppress negative emotions to maintain a perfect image, and this suppressive tendency may specifically interfere with the formation of PE satisfaction and its subsequent translation into psychological adaptation (Kuang and Feng, 2017). More generally, the interplay between perfectionism and motivational outcomes is sequentially mediated by self-efficacy and psychological resilience (Wan et al., 2023a), highlighting the need to examine how emotion regulation strategies, such as expressive suppression—may disrupt these positive chains within the PE context. Additionally, social role theory suggests that males face higher societal expectations regarding physical ability and athletic performance. Consequently, males high in concern over mistakes may perceive the PE environment as more threatening, leading to a more pronounced negative effect of concern over mistakes on satisfaction (Koivula, 1999; Ojio et al., 2026). Recent studies have also found gender differences in the relationship between perfectionism and satisfaction, with the negative effects of concern over mistakes being more prominent in males (Hill and Gotwals, 2025; Masson et al., 2003), This gender-specific pattern is not isolated to perfectionism: beyond perfectionism, gender has been shown to moderate the direct effect of parenting style on subjective well-being (Huang et al., 2022b), and subjective well-being itself can serve as a chain mediator in competence-related domains (Song et al., 2025). Taken together, these patterns justify our examination of gender as a first-stage moderator specifically on the concern over mistakes pathway in PE.

Based on the above analysis, the pathway mechanism through which the two perfectionism dimensions influence psychological adaptation via PE satisfaction has not been systematically tested, and the moderating roles of expressive suppression and gender in this process remain to be clarified. Therefore, integrating self-determination theory, emotion regulation theory, and social role theory, this study constructs a moderated mediation model to examine the transmission role of PE satisfaction between personal standards/concern over mistakes and subjective vitality, subjective well-being, and academic burnout, as well as the moderating effects of expressive suppression and gender on these mediating pathways. Collectively, the emerging literature across educational and occupational settings consistently demonstrates that psychological adaptation is shaped by cascading mechanisms involving domain-specific mediators (e.g., satisfaction, self-efficacy, coping) and moderators (e.g., resilience, emotion regulation, gender; Ding et al., 2024b; Wan et al., 2023b). These findings support the need for a context-sensitive, multivariate framework as proposed in this study.

This study hypothesizes:

H1: PE satisfaction mediates the relationship between perfectionism and psychological adaptation. Specifically, personal standards and concern over mistakes influence subjective vitality, subjective well-being, and academic burnout through PE satisfaction.

H2: Expressive suppression moderates the mediating pathway of perfectionism influencing psychological adaptation through PE satisfaction, and the moderation pattern differs across perfectionism dimensions.

H3: Gender moderates the mediating pathway of perfectionism influencing psychological adaptation through PE satisfaction, and the moderation effect differs across perfectionism dimensions.

**Figure 1:**
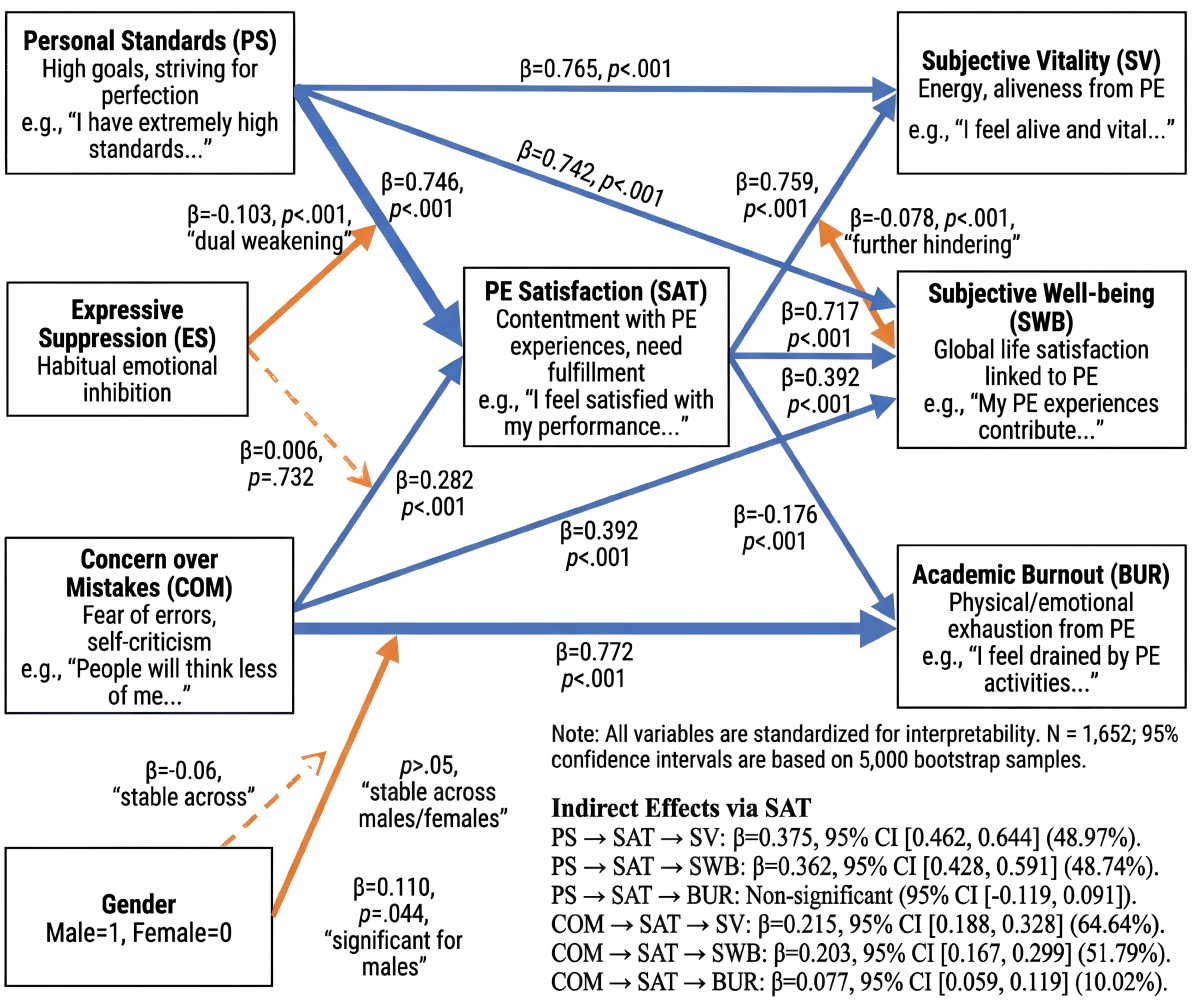
The moderated mediation model. Model Fit and Statistical Summary. CFA: *χ*^2^/df = 2.34, CFI = 0.92, RMSEA = 0.06, SRMR = 0.05. Sample: 1,652 Chinese college students (56.0% male, 44.0% female; M_age_ = 20.3, SD = 1.8). Controls: Gender and expressive suppression included as covariates.

The conceptual framework integrating these hypotheses is illustrated in Figure 1.

## Methods

### Participants

Using convenience sampling, undergraduate students were recruited from general education courses or PE theory courses at multiple universities in China. Sample size was determined *a priori* using G*Power 3. 1 software. Assuming a medium effect size (*f*^2^ = 0. 15), significance level *α* = 0.05, and statistical power of 0.95, the calculated minimum required sample size was 138. Considering potential invalid questionnaires and the need for subgroup comparisons, the target sample size was increased by approximately 85% to 2000 participants.

Recruitment was conducted during regular classroom sessions. After obtaining permission from course instructors, researchers briefly introduced the study content, explained its purpose, and emphasized the voluntary and anonymous nature of participation. All undergraduate students present were eligible. Interested students were provided with a link or QR code to the questionnaire on the Wenjuanxing platform. Two attention check items were embedded (e.g., “Please select ‘Agree’ for this item”). Upon completion, participants received 2 RMB as compensation.

Data collection yielded 2000 questionnaires. After excluding invalid questionnaires due to failed attention checks, missing data exceeding 10%, or completion time less than 10 min, 1,652 valid questionnaires were retained (effective response rate: 82.6%). Participants’ ages ranged from 17 to 24 years (M = 20.3, SD = 1.8). The sample comprised 925 males (56.0%) and 727 females (44.0%). Grade distribution was: 38.2% freshmen, 31.5% sophomores, 20. 1% juniors, and 10.2% seniors.

### Procedure

This study was approved by the Ethics Review Committee of the Department of Physical Education, Southeast University (Approval No: SEUE 2026) and adhered to the ethical principles of the Declaration of Helsinki. All participants read an informed consent form and clicked “Agree to Participate” before accessing the formal questionnaire.

Data collection occurred from March to May 2025. The survey was administered via the Wenjuanxing platform, compatible with both mobile and computer devices. In classroom settings, trained research assistants uniformly guided participants through questionnaire completion, ensuring independent responding without communication. Average completion time was 18 min (SD = 4.3). After questionnaire completion, researchers explained the study purpose to participants and provided contact information for any follow-up inquiries.

### Common method bias test

To examine potential common method bias, two methods were employed following Podsakoff et al.’s recommendations (Podsakoff et al., 2003). First, Harman’s single-factor test was conducted using exploratory factor analysis on all measurement items. Results showed that the first unrotated factor explained 30. 16% of the total variance, below the 50% critical threshold. Second, confirmatory factor analysis specifying a single-factor model yielded poor fit indices (χ^2^/df = 9.205, CFI = 0.669, TLI = 0.660, RMSEA = 0.100, SRMR = 0. 145). Both tests indicated that common method bias was not a serious concern in this study.

### Measures

*Perfectionism*: The Chinese version of the Frost Multidimensional Perfectionism Scale (FMPS), originally developed by Frost et al. (1990) and revised by Zi and Zhou (2006), was used. This 12-item scale comprises two dimensions: Concern over Mistakes (e.g., “People will probably think less of me if I make a mistake”) and Personal Standards (e.g., “I have extremely high standards for myself”). Items are rated on a 5-point Likert scale (1 = Strongly disagree, 5 = Strongly agree). In this study, Cronbach’s *α* for the total scale was 0.934, and for the two dimensions were 0.913 and 0.949, respectively.

*Emotion regulation strategy*: The Chinese version of the Emotion Regulation Questionnaire (ERQ-CRV), originally developed by Gross and John (2003) and revised by Wang et al. (2007), was used. This 10-item scale comprises two dimensions: Cognitive Reappraisal (6 items) and Expressive Suppression (4 items). Items are rated on a 7-point Likert scale (1 = Strongly disagree, 7 = Strongly agree). In this study, Cronbach’s *α* for Cognitive Reappraisal and Expressive Suppression were 0.932 and 0.864, respectively.

*PE satisfaction*: The Physical Education Course Satisfaction Scale for Chinese College Students (PECSS-CCS), developed by Jin and Xi (2026), was used. This 26-item scale comprises six dimensions: Teaching Characteristics, Teacher Characteristics and Attitude, Course Experience, Learning Atmosphere, Facilities and Equipment, and Assessment Methods. Items are rated on a 5-point Likert scale (1 = Strongly disagree, 5 = Strongly agree). The PECSS-CCS has been formally published in Frontiers in Public Health. In the present study, Cronbach’s *α* for the total scale was 0.926. has six-factor structure and good internal consistency (Cronbach‘s *α* ranging from 0.866 to 0.953 across subscales).

*Subjective well-being*: The Chinese version of the Subjective Happiness Scale (SHS-C), originally developed by Lyubomirsky and Lepper (1999) and revised by Nan et al. (2014), was used. This 4-item scale measures individuals’ global subjective evaluation of their happiness. Items are rated on a 7-point Likert scale (1 = Strongly disagree, 7 = Strongly agree). In this study, Cronbach’s *α* was 0.867.

*Subjective vitality*: The Chinese version of the Subjective Vitality Scale (SVS), originally developed by Ryan and Frederick (1997) and revised by Cheng and Wong (2025), was used. This 5-item scale (e.g., “I feel alive and vital”) measures subjective vitality. Items are rated on a 7-point Likert scale (1 = Not true at all, 7 = Very true). In this study, Cronbach’s *α* was 0.928.

### Data analysis

Data analysis was conducted using SPSS 26.0, AMOS 26.0, and the PROCESS 4. 1 macro (Hayes, 2013). First, confirmatory factor analysis (CFA) was performed using AMOS 26.0 to test the discriminant and convergent validity of the five latent variables: Personal Standards, Concern over Mistakes, PE Satisfaction, Subjective Vitality, and Subjective Well-being. Subsequently, descriptive statistics and Pearson correlations were calculated using SPSS 26.0 to examine means, standard deviations, and relationships among variables. Next, PROCESS Model 4 was used to test the mediating effects of PE satisfaction in the relationships between the two perfectionism dimensions (Personal Standards, Concern over Mistakes) and the three psychological adaptation indicators (Subjective Vitality, Subjective Well-being, Academic Burnout). Bias-corrected bootstrap (5,000 samples) was used to estimate 95% confidence intervals for indirect effects. Then, PROCESS Model 58 was employed to examine the moderating effects of Expressive Suppression on the first stage (perfectionism to PE satisfaction) and second stage (PE satisfaction to psychological adaptation) of the mediating pathways. Conditional indirect effects were calculated to reveal changes in mediation effects across different levels of Expressive Suppression. Finally, PROCESS Model 59 was used to test the moderating effect of gender on the entire mediating pathway, calculating conditional indirect effects for male and female groups separately. Gender was controlled as a covariate or treated as a moderator in respective analyses. Significance level was set at *α* = 0.05.

## Results

### Descriptive statistics and correlation analysis

Means, standard deviations, and Pearson correlation coefficients for all variables are presented in Table 1. Following the conceptual sequence of the study, correlations are reported in order of the two perfectionism dimensions’ associations with other variables. Personal Standards showed a strong positive correlation with PE Satisfaction (*r* = 0.746, *p* < 0.01), Subjective Vitality (*r* = 0.765, *p* < 0.01), and Subjective Well-being (*r* = 0.742, *p* < 0.01), and a moderate positive correlation with Academic Burnout (*r* = 0.632, *p* < 0.01) and Concern over Mistakes (*r* = 0.632, *p* < 0.01). Concern over Mistakes showed a strong positive correlation with Academic Burnout (*r* = 0.773, *p* < 0.01), a moderate positive correlation with Personal Standards (*r* = 0.632, *p* < 0.01) and Subjective Well-being (*r* = 0.392, *p* < 0.01), a moderate-to-weak positive correlation with Subjective Vitality (*r* = 0.332, *p* < 0.01), and a weak positive correlation with PE Satisfaction (*r* = 0.282, *p* < 0.01). The positive associations between Concern over Mistakes and both Subjective Vitality and Subjective Well-being, though unexpected from a purely maladaptive perspective, suggest that Concern over Mistakes possesses a dual nature encompassing both crisis and opportunity-a finding examined further in the Discussion. PE Satisfaction was significantly positively correlated with Personal Standards (*r* = 0.746, *p* < 0.01), Subjective Vitality (*r* = 0.759, *p* < 0.01), and Subjective Well-being (*r* = 0.717, *p* < 0.01), and significantly negatively correlated with Academic Burnout (*r* = −0.176, *p* < 0.01), indicating that students more satisfied with their PE experience exhibited higher levels of psychological adaptation. Expressive Suppression was significantly negatively correlated with PE Satisfaction (*r* = −0.081, *p* < 0.05), Subjective Vitality (*r* = −0.129, *p* < 0.01), and Subjective Well-being (*r* = −0.104, *p* < 0.01), indicating that habitual emotional suppression may hinder college students’ ability to benefit from PE courses. Following Cohen's (1988) conventions, correlation coefficients were interpreted as: small (|*r*| > = 0.10), medium (|*r*| > = 0.30), and large (|*r*| > = 0.50).

**Table 1:** Descriptive statistics and correlation matrix.

Variable	M	SD	1	2	3	4	5	6
1. PE satisfaction	3.58	0.98	1					
2. Personal standards	3.41	0.94	0.746**	1				
3 Concern over mistakes	3.11	1.13	0.282**	0.632**	1			
4. Subjective vitality	4.57	1.40	0.759**	0.765**	0.332**	1		
5. Subjective well-being	4.57	1.36	0.717**	0.742**	0.392**	0.842**	1	
6. Academic burnout	4.31	1.36	−0.176**	−0.632**	−0.773**	−0.277**	−0.324**	1
7. Expressive suppression	4.25	1.44	−0.081*	0.150**	0.130**	−0.129**	−0.104**	0.188**

*N* = 1,652; *p < 0.05, **p < 0.01.

### Mediation analysis

PROCESS Model 4 (Hayes, 2013) was used to test the mediating role of PE Satisfaction in the relationship between perfectionism and psychological adaptation, controlling for gender. Bootstrap resampling with 5,000 samples was used to calculate bias-corrected confidence intervals for indirect effects. Results are summarized in Table 2.

**Table 2:** Summary of mediation analysis.

Path	Total effect B(SE)	Total effect *β*	Indirect effect B(SE)	Indirect effect *β*	95% CI	Direct effect B(SE)	Direct effect *β*	Mediation proportion
PS → SAT → SV	1.131 (0.033)**	0.765	0.554 (0.046)**	0.375	[0.462, 0.644]	0.577 (0.040)**	0.390	48.97%
PS → SAT → SWB	1.046 (0.033)**	0.742	0.510 (0.041)**	0.362	[0.428, 0.591]	0.536 (0.040)**	0.381	48.74%
PS → SAT → BUR	0.888 (0.032)**	0.632	−0.018 (0.053)	−0.013	[−0.119, 0.091]	0.906 (0.044)**	0.645	0%
COM → SAT → SV	0.397 (0.040)**	0.332	0.257 (0.036)**	0.215	[0.188, 0.328]	0.140 (0.026)**	0.118	64.64%
COM → SAT → SWB	0.446 (0.037)**	0.392	0.231 (0.033)**	0.203	[0.167, 0.299]	0.216 (0.026)**	0.189	51.79%
COM → SAT → BUR	0.870 (0.025)**	0.772	0.087 (0.016)**	0.077	[0.059, 0.119]	0.783 (0.026)**	0.695	10.02%

PS = personal standards, COM = concern over mistakes, SAT = PE satisfaction, SV = subjective vitality, SWB = subjective well-being, BUR = academic burnout. Bootstrap 5,000 samples; **p < 0.01. Mediation proportions are descriptive only; given the cross-sectional design, they should not be interpreted as causal shares.

*Personal standards pathway*: Personal standards significantly positively predicted subjective vitality (Total effect: B = 1.131, SE = 0.033, *t* = 33.96, *p* < 0.001, *β* = 0.765) and Subjective Well-being (Total effect: B = 1.046, SE = 0.033, *t* = 31.71, *p* < 0.001, *β* = 0.742). The indirect effects via PE Satisfaction were significant for both outcomes. For vitality, the indirect effect was B = 0.554, *β* = 0.375, 95% CI [0.462, 0.644], representing approximately 48.97% of the total effect. For well-being, the indirect effect was B = 0.510, *β* = 0.362, 95% CI [0.428, 0.591], representing approximately 48.74% of the total effect. The indirect effect of Personal Standards on Academic Burnout via PE.

Satisfaction was not significant, 95% CI [−0.119, 0.091]. This indicates that PE Satisfaction is an important mechanism through which Personal Standards translate into positive psychological adaptation, but it does not explain the relationship between Personal Standards and academic burnout.

*Concern over mistakes pathway*: Concern over mistakes significantly predicted subjective vitality (Total effect: B = 0.397, SE = 0.040, *t* = 9.92, *p* < 0.001, *β* = 0.332), Subjective Well-being (Total effect: B = 0.446, SE = 0.037, *t* = 11.98, *p* < 0.001, *β* = 0.392), and Academic Burnout (Total effect: B = 0.870, SE = 0.025, *t* = 34.34, *p* < 0.001, *β* = 0.772). The indirect effects via PE Satisfaction were significantly positive for vitality (B = 0.257, *β* = 0.215, 95% CI [0.188, 0.328], descriptive mediation proportion 64.64%) and well-being (B = 0.231, *β* = 0.203, 95% CI [0.167, 0.299], descriptive mediation proportion51.79%). The indirect effect on Academic Burnout, while significant, was weak (B = 0.087, *β* = 0.077, 95% CI [0.059, 0. 119], descriptive mediation proportion10.02%).

### Moderating effect of expressive suppression

PROCESS Model 58 was used to test the moderating effect of expressive suppression on the first stage (from perfectionism to satisfaction) and second stage (from satisfaction to psychological adaptation) of the mediating pathways, controlling for gender. *R*^2^ and *F* values for each regression model are shown below Table 3. Conditional indirect effects were calculated based on 5,000 bootstrap samples.

**Table 3:** Moderating effect of expressive suppression on mediating pathways (model 58).

Path	First Stage interaction B(SE)	First stage *β*	Second stage interaction B(SE)	Second stage *β*	Conditional indirect effect (low/med/high ES)
PS → SAT → SV	−0.103 (0.015)**	−0.103	−0.007 (0.019)	−0.007	0.579/0.475/0.374
PS → SAT → SWB	−0.103 (0.015)**	−0.103	−0.078 (0.019)**	−0.078	0.546/0.379/0.243
COM → SAT → SV	0.006 (0.018)	0.006	−0.013 (0.020)	−0.013	−0.135/−0.124/−0.114
COM → SAT → SWB	0.006 (0.018)	0.006	−0.081 (0.020)**	−0.081	0.194/0.168/0.137

**p < 0.01; Conditional indirect effects based on 5,000 bootstrap samples, all confidence intervals exclude zero. Regression model *R*^2^ and *F* values: For PS pathways, Sat equation *R*^2^ = 0.584, *F*(4, 1,647) = 287.56, *p* < 0.001; *SV* equation *R*^2^ = 0.695, *F*(5, 1,646) = 373.83, *p* < 0.001; SWB equation *R*^2^ = 0.671, *F*(5, 1,646) = 334.80, *p* < 0.001. For COM pathways, Sat equation *R*^2^ = 0.305, *F*(4, 1,647) = 89.88, *p* < 0.001; SV equation *R*^2^ = 0.648, *F*(5, 1,646) = 301.37, *p* < 0.001; SWB equation *R*^2^ = 0.646, *F*(5, 1,646) = 299.66, *p* < 0.001*.

**Figure 2:**
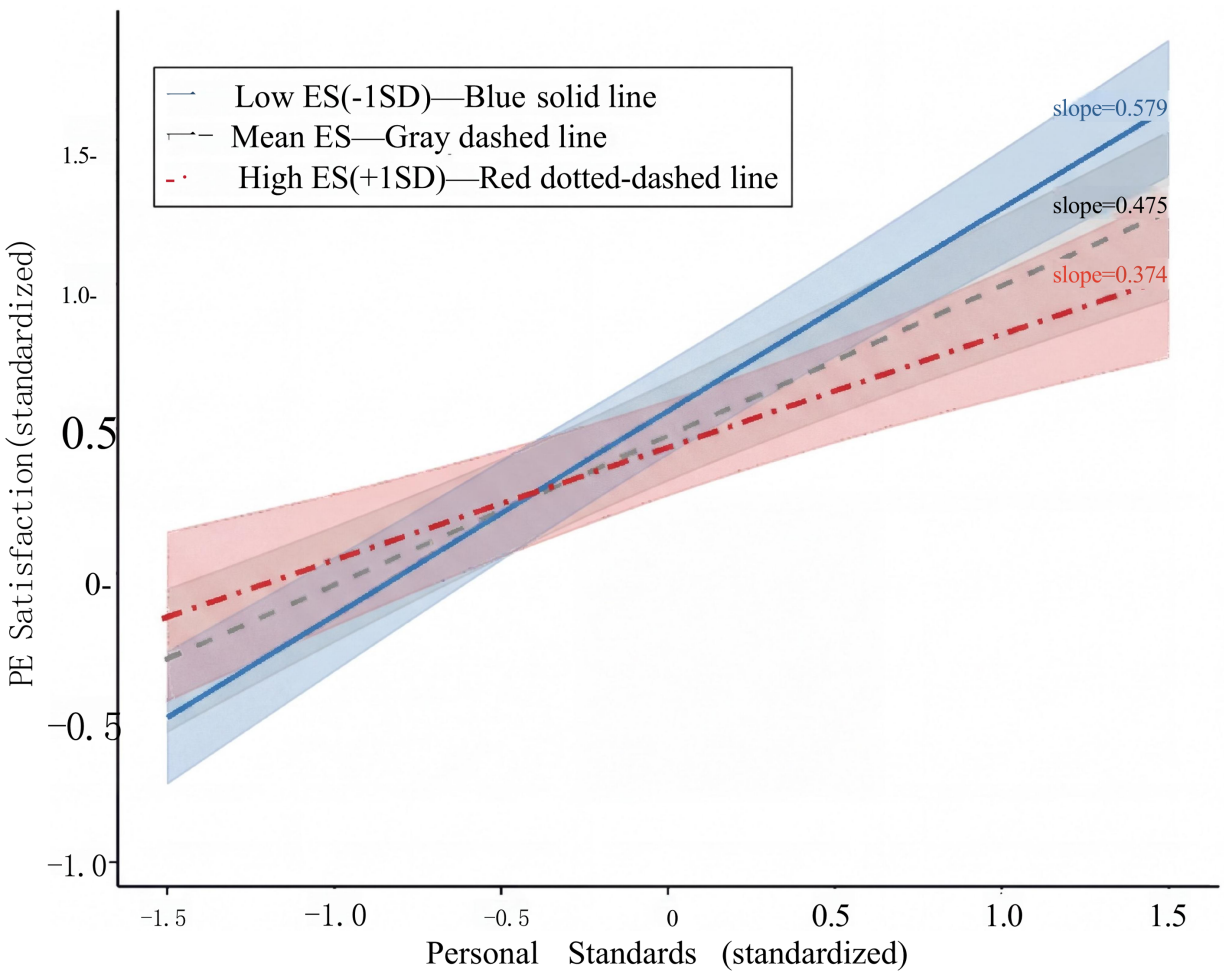
Moderating effect of expressive suppression on the relationship between personal standards and PE satisfaction. All variables are standardized. The figure displays the conditional effect of personal standards (PS) on PE satisfaction (SAT) at low(−1SD), mean, and high(+1SD) levels of expressive suppression(ES). Shaded areas represent 95%confidence intervals. The regression equation for SAT is: SAT − 0.577 + 0.765 × PS − 0, 103 × ES − 0.103 × (PS × ES). The interaction term is significant (B = −0.103, *p* < 0.001). Simple slopes for PS→SAT are 0.579 (low ES), 0.475 (mean ES), and 0.374(high ES). Conditional indirect effects of PS on subjective vitality via SAT (not shown) decrease from 0.579 to 0.374 across ES levels. *N* = 1,652; *α* =0.05.

**Figure 3:**
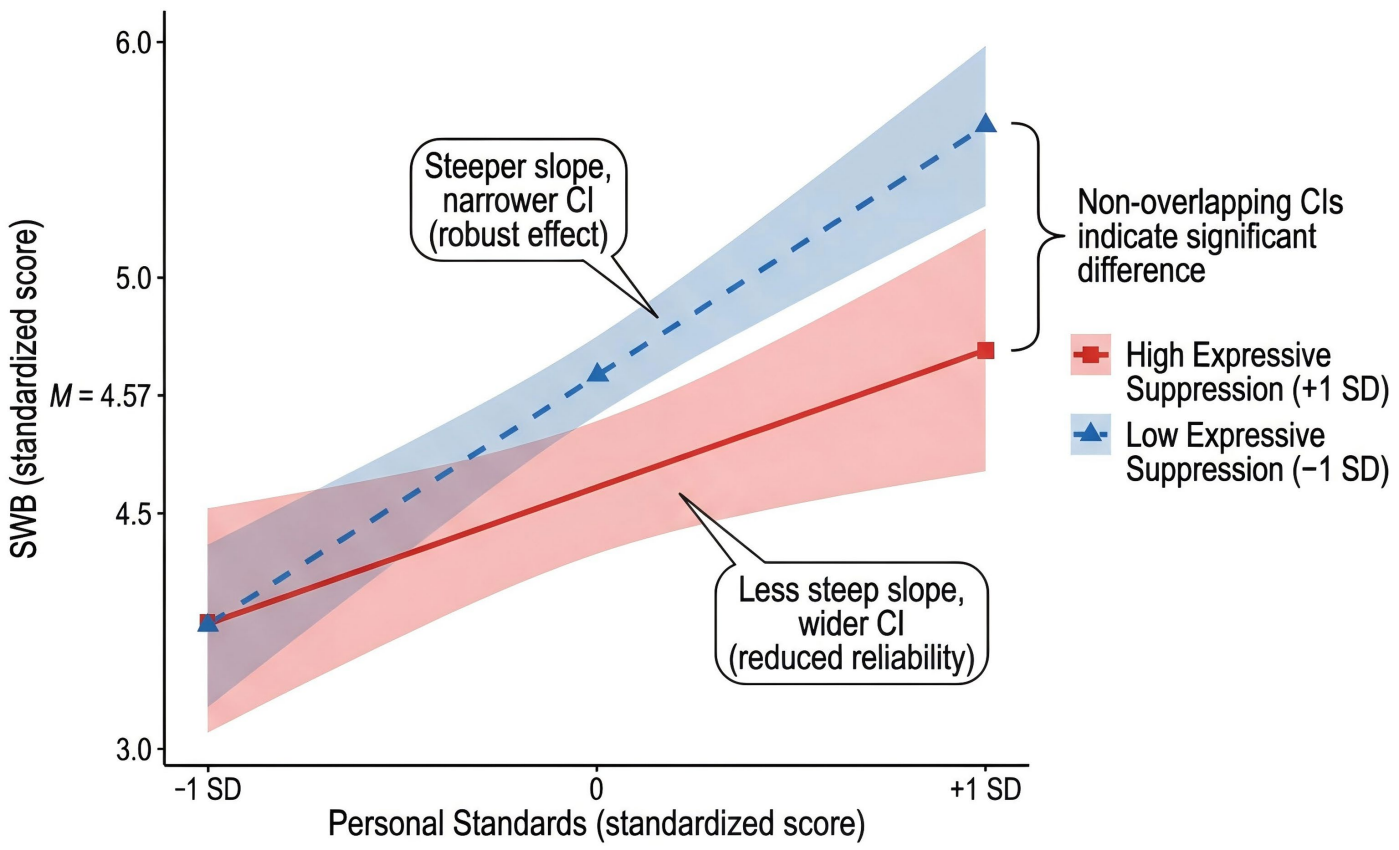
Moderating role of expressive suppression in the pathway from personal standards to well-being through PE satisfaction. *Statistical summary*: regression equations: PE satisfaction = 0.577 + 0.765 × Personal Standards − 0.103 × Expressive Suppression. SWB = 0.900 + 0.536 × PE Satisfaction − 0.078 × Expressive Suppression −0.007 × (Personal Standards × Expressive Suppression) Interaction effects: First stage (Personal Standards→PE Satisfaction): *β* = −0.103, *p* < 0.001 (dual weakening of the positive link). Second stage (PE Satisfaction→SWB): *β* = −0.078, *p* < 0.001 (further suppression of satisfaction's benefits). Total indirect effect: Reduced by 55.5% at high vs. low suppression (0.546→0.243) (figure generated using R ggplot2; *N* = 1,652; *α* = 0.05).

*Personal standards pathway*: For the pathway from personal standards to subjective vitality via PE satisfaction, Figure 2 displays the moderating effect of expressive suppression on the relationship between personal standards and PE satisfaction. Expressive Suppression significantly negatively moderated the first stage (B = −0.103, SE = 0.015, *t* = −6.73, *p* < 0.001, *β* = −0. 103), but the moderation on the second stage was not significant (B = −0.007, SE = 0.019, *t* = −0.38, *p* = 0.708, *β* = −0.007). Conditional indirect effects decreased with increasing levels of Expressive Suppression: 0.579, 0.475, and 0.374 at low, medium, and high levels of Expressive Suppression, respectively, with all confidence intervals excluding zero. For the pathway from personal standards to subjective well-being via PE satisfaction, as shown in Figure 3. Expressive suppression significantly negatively moderated both stages (first stage *β* = −0.103, *p* < 0.001; second stage *β* = −0.078, *p* < 0.001). Conditional indirect effects decreased from 0.546 (low ES) to 0.243 (high ES), a reduction of 55.5%. This indicates that Expressive Suppression not only hinders the translation of Personal Standards into satisfaction but also further interferes with the enhancement of well-being through satisfaction.

*Concern over mistakes pathway*: For the pathway from concern over mistakes to subjective vitality via PE satisfaction, the moderating effects of expressive suppression were not significant (first stage *β* = 0.006, *p* = 0.732; second stage *β* = −0.013, *p* = 0.522). Conditional indirect effects at low, medium, and high Expressive Suppression were −0.135, −0. 124, and −0. 114, respectively, with all confidence intervals excluding zero. For the pathway from concern over mistakes to subjective well-being via PE satisfaction, Expressive Suppression significantly negatively moderated the second stage (*β* = −0.081, *p* < 0.001), while the first stage moderation was not significant (*p* = 0.732). Conditional indirect effects decreased from 0.194 at low Expressive Suppression to 0.137 at high Expressive Suppression, indicating that high Expressive Suppression attenuates the positive indirect effect of Concern over Mistakes on well-being via satisfaction.

### Moderating effect of gender

PROCESS Model 59 was used to test the moderating effect of gender on the mediating pathways, controlling for Expressive Suppression. *R*^2^ and *F* values for each regression model are shown below Table 4. Conditional indirect effects were calculated based on 5,000 bootstrap samples.

**Table 4:** Moderating Effect of Gender on Mediating Pathways (Model 59).

Path	First stage interaction B(SE)	First stage *β*	Second stage interaction B(SE)	Second stage *β*	Conditional indirect effect (males/females)
PS → SAT → SV	0.057 (0.052)	0.057	0.041 (0.088)	0.041	Males: 0.492 [0.372, 0.622] Female: 0.561 [0.423, 0.696]
PS → SAT → SWB	0.057 (0.052)	0.057	0.059 (0.088)	0.059	Male: 0.438 [0.331, 0.551] Female: 0.517 [0.397, 0.646]
COM → SAT → SV	0.110 (0.055)*	0.110	0.083 (0.064)	0.083	Male: −0.157 [−0.239, −0.077] Female: −0.062 [−0.160, 0.045]
COM → SAT → SWB	0.110 (0.055)*	0.110	0.079 (0.062)	0.079	Male: −0.134 [−0.203, −0.066] Female: −0.053 [−0.139, 0.038]

*p < 0.05; Brackets contain 95% confidence intervals; Bootstrap 5,000 samples. Regression model *R*^2^ and *F* values: For PS pathways, Sat equation *R*^2^ = 0.561, *F*(4, 1,647) = 262.47, *p* < 0.001; SV equation *R*^2^ = 0.696, *F*(6, 1,645) = 312.74, *p* < 0.001; SWB equation *R*^2^ = 0.666, *F*(6, 1,645) = 272.28, *p* < 0.001. For COM pathways, Sat equation *R*^2^ = 0.308, *F*(4, 1,647) = 91.30, *p* < 0.001; SV equation *R*^2^ = 0.651, *F*(6, 1,645) = 254.48, *p* < 0.001; SWB equation *R*^2^ = 0.642, *F*(6, 1,645) = 245.10, *p* < 0.001*.

*Personal standards pathway*: All interaction terms were non-significant (*p* > 0.05). Conditional indirect effects were significant for both males and females, with similar effect sizes: For the vitality pathway, male indirect effect = 0.492 [0.372, 0.622], female = 0.561 [0.423, 0.696]; for the well-being pathway, male = 0.438 [0.331, 0.551], female = 0.517 [0.397, 0.646]. This indicates that the process through which Personal Standards influences psychological adaptation via PE Satisfaction is stable across genders.

*Concern over mistakes pathway*: For the pathway from concern over mistakes to subjective vitality via PE satisfaction, the first-stage interaction was significant (B = 0.110, SE = 0.055, *t* = 2.02, *p* = 0.044, *β* = 0. 110), while the second-stage interaction was not significant (*β* = 0.083, *p* = 0. 199). The conditional indirect effect was significantly negative for males (−0.157, 95% CI [−0.239, −0.077], *β* = −0. 13) but non-significant for females (−0.062, 95% CI [−0.160, 0.045], *β* = −0.05). For the COM to SAT to SWB pathway, similarly, the first-stage interaction was significant (*β* = 0.110, *p* = 0.044), while the second-stage interaction was not significant (*β* = 0.079, SE = 0.062, *t* = 1.27, *p* = 0.205). The conditional indirect effect was significantly negative for males (−0.134, 95% CI [−0.203, −0.066], *β* = −0. 12) but non-significant for females (−0.053, 95% CI [−0.139, 0.038], *β* = −0.05).

This contrast reveals that the process through which Concern over Mistakes impairs psychological adaptation by reducing PE Satisfaction exists only in males; females appear “immune” to this effect.

## Discussion

This study investigated the internal mechanisms through which college students’ perfectionistic personality influences psychological adaptation within the PE context using a moderated mediation model. We found that PE Satisfaction transmitted nearly half of the effect of Personal Standards on positive adaptation but barely buffered the relationship between Concern over Mistakes and Academic Burnout. Expressive suppression exhibited differential moderating effects on these two pathways, manifesting as “dual weakening” for the Personal Standards pathway and “emotional blunting” for the Concern over Mistakes pathway. Gender only moderated the Concern over Mistakes pathway and exclusively affected its first stage, the process through which Concern over Mistakes impairs adaptation by reducing satisfaction was significant only in males. These findings suggest that the “crisis” and “opportunity” of perfectionism are not inherent attributes of the personality trait but rather products dynamically constructed through the interaction of contextual features, emotional processing styles, and social positions.

PE Satisfaction explained nearly half of the effect between Personal Standards and both subjective vitality (indirect effect B = 0.554, 95% CI [0.462, 0.644], 48.97%) and subjective well-being (B = 0.510, 95% CI [0.428, 0.591], 48.74%). This aligns closely with self-determination theory (Ryan and Deci, 2000): PE class, as an embodied competence-validation setting, provides a vehicle for individuals with high Personal Standards to satisfy basic psychological needs, and this domain-specific satisfaction spills over into daily psychological energy, becoming a significant source of positive adaptation (Vazou et al., 2019). In other words, the positive effects of personality traits do not manifest automatically but depend on suitable contextual carriers: embodied competence-validation settings are crucial conditions for Personal Standards to yield psychological gains (Frisanco et al., 2022). Notably, the indirect effect of Personal Standards on Academic Burnout was not significant (B = −0.018, CI [−0.119, 0.091]), indicating that PE Satisfaction is not the pathway through which Personal Standards influences burnout. Consistent with Gaudreau’s findings among 376 graduate students, perfectionistic strivers reported higher research performance satisfaction but, after controlling for satisfaction, still experienced higher levels of academic burnout and dropout intentions (Gaudreau and Benoît, 2025).

For Concern over Mistakes, PE Satisfaction similarly transmitted a substantial proportion of positive effects: the indirect effect on vitality was B = 0.257 (CI [0.188, 0.328]), descriptive mediation proportion64.64%, and on well-being was B = 0.231 (CI [0.167, 0.299]), descriptive mediation proportion51.79%. However, this indirect effect on Academic Burnout was minimal (B = 0.087, CI [0.059, 0. 119]), representing only one-tenth of Concern over Mistakes’ total effect (B = 0.870). This finding challenges the traditional view of Concern over Mistakes as a purely maladaptive trait (Stoeber, 2018a), demonstrating its psychological effects are contextually malleable: In the composite context of PE, which simultaneously involves evaluative pressure and competence experiences, positive course experiences can partially mitigate fear of failure, allowing individuals high in Concern over Mistakes to also benefit. However, this contextual gain cannot eliminate their inherent burnout tendency—which operates primarily through a direct pathway. This aligns with research by González-Cutre et al. (2025). The unexpectedly positive correlations between concern over mistakes and both subjective vitality (*r* = 0.332) and subjective well-being (*r* = 0.392) warrant careful examination. Three mechanisms may account for this adaptive concern over mistakes phenomenon. First, in the PE context, moderate concern over mistakes may motivate students to invest greater effort in skill development and physical preparation, leading to genuine competence gains and subsequent well-being enhancement-a process consistent with the challenge-threat framework in performance psychology. Second, within collectivistic educational environments such as China, concern over mistakes may partially reflect a socially valued orientation toward self-improvement and responsibility to group standards, attenuating its purely maladaptive effects. Third, the substantial indirect effects via PE satisfaction (64.64% for vitality, 51.79% for well-being) suggest that the positive experience derived from PE participation can partially transform the motivational energy of concern over mistakes into adaptive outcomes, even though this transformation is less efficient than for personal standards. However, this adaptive potential has clear boundaries: the minimal buffering of academic burnout (10.02%) indicates that concern over mistakes maintains an independent and robust burnout-generating pathway that contextual gains cannot override. This pattern suggests that concern over mistakes is best conceptualized not as uniformly maladaptive but as contextually plastic-its ultimate psychological consequences depend on the balance between competence-gain mechanisms and evaluative-threat mechanisms activated within specific settings.

Previous research may have failed to reveal this complexity due to a narrow focus on single contexts. Most studies have focused on private settings like academic courses, which can activate evaluation fears but cannot provide embodied competence experiences (Bergstrom et al., 2011). The uniqueness of PE lies in its simultaneous activation of perfectionists’ achievement strivings and evaluative fears (Hall et al., 1998; Klein et al., 2020). The interplay between these two psychological processes makes the ultimate outcome highly dependent on the outcome indicator: For positive indicators, competence gains sufficiently offset fears; for Academic Burnout, a long-term negative accumulation, the trait’s inherent cognitive tendencies prevail (Barkoukis et al., 2024; Stoeber and Becker, 2008). This result advances perfectionism research from “trait determinism” toward “trait-context interactionism” and offers a new conceptualization of contextual factors: PE Satisfaction functions as a “gain system” rather than a “buffer system,”it amplifies the effects of positive traits and provides additional positive resources for maladaptive traits, but cannot fundamentally alter the trait’s inherent adaptive tendencies.

The moderation by expressive suppression on the two perfectionism pathways exhibited a striking functional reversal. On the Personal Standards pathway, expressive suppression weakened both the first stage (Personal Standards to Satisfaction, B = −0.103, *p* < 0.001) and the second stage (Satisfaction to well-being, B = −0.078, *p* < 0.001), reducing the indirect effect of Personal Standards on well-being via satisfaction from 0.546 at low expressive suppression to 0.243 at high expressive suppression—a 55.5% reduction. This represents a classic “dual weakening.” In contrast, on the Concern over Mistakes pathway, expressive suppression had no significant effect on the first stage (B = 0.006, *p* = 0.732) but significantly negatively moderated the second stage (B = −0.081, *p* < 0.001), causing the negative indirect effect of Concern over Mistakes on well-being via low satisfaction to diminish from 0.194 to 0. 137: an “emotional blunting” effect (Seligowski et al., 2015).

This result engages in deep dialogue with emotion regulation theory (Gross, 1998). The theory posits that expressive suppression consumes cognitive resources and hinders the internalization of positive emotions, which well explains its “dual weakening” effect on the Personal Standards pathway: Individuals high in Personal Standards who also employ high expressive suppression struggle both to translate their goal pursuit into satisfying course experiences and to fully internalize existing positive experiences (Kalokerinos et al., 2015; Li et al., 2020). However, the theory implicitly assumes that the effects of emotion regulation strategies are fixed, labeling expressive suppression as a “maladaptive strategy,” while neglecting the interactive plasticity between strategies and personality traits (Eldesouky and English, 2023). This study demonstrates that the function of the same strategy can differ dramatically depending on the personality dimension it interacts with: For those striving for excellence, expressive suppression is an “impediment to gain”; for those fearing failure, it becomes an “anesthetic for pain,”by blunting awareness of negative emotions, it temporarily blocks the transmission from low satisfaction to reduced well-being. However, this “adaptiveness” comes at the cost of authentic emotional experience. In East Asian cultures emphasizing emotional restraint, expressive suppression also carries specific social normative functions (Koydemir and Essau, 2017). The “emotional blunting” effect observed here may reflect the cultural adaptability of the strategy. However, this interpretation is speculative given that the present study did not directly measure cultural values; direct testing through cross-cultural comparative research is needed. From a cognitive resource perspective, the dual weakening on the personal standards pathway can be understood as a resource depletion process: individuals high in personal standards already invest substantial cognitive resources in pursuing ambitious goals; adding expressive suppression demands further depletes the self-regulatory capacity needed to fully engage with and benefit from PE experiences (Muraven and Baumeister, 2000). The first-stage weakening (beta = −0.103) may reflect suppression-induced interference with the encoding of competence feedback, while the second-stage weakening (beta = −0.078) may reflect reduced capacity for the elaboration and internalization of positive experiences that have already been registered. This dual-process interference makes expressive suppression particularly costly for individuals whose psychological gains depend on actively processing and internalizing domain-specific positive experiences.

Gender moderation of the mediating pathways exhibited strict dual specificity: it operated only on the Concern over Mistakes pathway and only affected the first stage (Concern over Mistakes to Satisfaction). The interaction between Concern over Mistakes and gender was significant (B = 0.110, *p* = 0.044), with conditional indirect effects showing that the process of Concern over Mistakes impairing adaptation via reduced satisfaction was significantly negative for males (vitality pathway indirect effect = −0.157, 95% CI [−0.239, −0.077]; well-being pathway = −0.134, 95% CI [−0.203, −0.066]), but completely disappeared for females (vitality = −0.062, CI [−0.160, 0.045]; well-being = −0.053, CI [−0.139, 0.038]). No gender interactions were found on the Personal Standards pathway, nor were there gender differences in the second stage.

From a cognitive resource consumption perspective, males high in concern over mistakes may expend disproportionate cognitive resources on monitoring and suppressing error-related distress during PE, reducing the attentional and emotional resources available for deriving satisfaction from the activity itself-a pathway consistent with ego-depletion models of self-regulation. Furthermore, the specificity of gender moderation to the first stage (concern over mistakes to satisfaction) rather than the second stage (satisfaction to adaptation) carries an important implication: once satisfaction is formed, its spillover benefits to well-being operate similarly across genders, suggesting that interventions should target the initial perception and evaluation of PE experiences rather than the downstream well-being processes. This first-stage specificity also rules out alternative explanations based on gender differences in emotional reactivity or well-being set points, as such differences would manifest across all pathways rather than selectively in the concern over mistakes to satisfaction link.

This pattern does not stem from gender differences in levels of Concern over Mistakes (no significant mean difference in this study) but rather from the selective amplification by social role expectations (Koivula, 1999; Lundqvist et al., 2024). Through long-term socio-cultural construction, physical ability and athletic performance have become important markers of male gender identity, making PE class a significant arena for males to validate their gender role (Xiong and Li, 2024). This subjects males to a unique “failure-avoidance pressure” in PE—for them, avoiding public mistakes is more urgent than striving for excellence. The core of Concern over Mistakes is excessive worry about failure and negative evaluation; the superposition of these factors leads to heightened activation of males’ concern over mistakes in PE, thereby impairing course satisfaction (Frost et al., 1995). For females, athletic performance is not central to their gender identity, rendering PE class less threatening and thus nullifying the negative effects of Concern over Mistakes (Sher et al., 2025). The absence of gender differences in the second stage further confirms the cross-gender stability of satisfaction’s spillover effects (Kim-Prieto et al., 2020).

This finding liberates explanations of gender differences from attributions of “intrinsic male–female differences” and instead points to a social constructionist logic: Gender differences in personality trait effects arise not from inherent psychological disparities between men and women, but from the different meanings that socio-cultural contexts give the same situation (Eagly, 1987, 2000). It advances gender differences research from superficial mean comparisons toward deep deconstruction of social construction.

Synthesizing these findings, the psychological effects of personality traits do not exist in isolation but are shaped through the triple interaction of contextual features, emotional processing styles, and social roles. Contextual features (e.g., the embodiment of PE) activate specific dimensions of personality, enabling their effects to manifest; emotional processing styles (e.g., expressive suppression) modulate the connection between personality and contextual experience, determining whether experiences can be internalized; social roles (e.g., gender) imbue contexts with different meanings, selectively amplifying or suppressing certain personality effects. The dynamic interplay among these three factors ultimately determines whether a personality trait manifests as “crisis” or “opportunity.”

This framework suggests that when studying any personality trait, we must ask three questions: In what context is it activated? How does the individual’s emotional processing intervene? How do social roles give meaning to this process?

Several limitations should be noted. First, the cross-sectional design limits causal inference. While the moderated mediation model reveals patterns of association among variables, the dynamic relationships between perfectionism, PE Satisfaction, and psychological adaptation require longitudinal validation. Second, the sample was drawn solely from universities in Jiangsu Province; convenience sampling may affect external validity. Future research should test model stability across broader geographic regions and cultural contexts. Third, the current study did not collect ethnicity information, as the sample predominantly consisted of Han Chinese students; nevertheless, future research should consider ethnic diversity where applicable. Additionally, students’ major distribution (e.g., arts, science/engineering, physical education) was not recorded in the original survey, which represents a limitation. Different professional backgrounds may lead to varying perceptions of PE courses, and this potential confounding variable should be addressed in future research through stratified sampling across academic disciplines. Fourth, all data were self-reported. Although statistical tests indicated common method bias was within acceptable limits, incorporating multi-source data (e.g., teacher evaluations, physiological indicators) would enhance objectivity. Finally, the “emotional blunting” effect of expressive suppression may appear protective in the short term, but its long-term costs remain unclear. Future research combining longitudinal designs and experimental paradigms should examine whether this strategy cumulatively leads to emotion regulation disorders over time.

## Conclusion

This study reveals that the “crisis” and “opportunity” of perfectionism are not inherent attributes, but rather dynamic products shaped through activation by the PE context, processing by emotion regulation strategies, and selective amplification by social role expectations. PE satisfaction functions as a “gain system,” capable of transforming the achievement drive of personal standards into nearly half of positive psychological capital, yet unable to buffer the inherent academic burnout tendency of concern over mistakes. Expressive suppression exhibits functionally reversed moderating effects on the two pathways—“dual weakening” on the personal standards pathway and “emotional blunting” on the concern over mistakes pathway—challenging the traditional dichotomous view of emotion regulation strategies as inherently “good” or “bad.” Gender moderates only the concern over mistakes pathway and exclusively in males, indicating that differences stem from social role expectations rather than essential psychological disparities.

## Data Availability

The original contributions presented in the study are included in the article/supplementary material, further inquiries can be directed to the corresponding authors.
